# Effectiveness of Nutritional Guidance Focusing on Leucine Intake During Cardiac Rehabilitation Maintenance

**DOI:** 10.14789/jmj.JMJ23-0008-OA

**Published:** 2023-12-22

**Authors:** SAKI KAWAKUBO, KOSUKE FUKAO, YUKI SOMEYA, JUNYA NISHIMURA, MAYUMI DOI, YUSEI SATO, MIHO YOKOYAMA, MINORU TABATA, TOHRU MINAMINO, HISASHI NAITO

**Affiliations:** 1 Graduate School of Health and Sports Science, Juntendo University, Chiba, Japan; 1 Graduate School of Health and Sports Science, Juntendo University, Chiba, Japan; 2 Department of Cardiovascular Biology and Medicine, Juntendo University Graduate School of Medicine, Tokyo, Japan; 2 Department of Cardiovascular Biology and Medicine, Juntendo University Graduate School of Medicine, Tokyo, Japan; 3 Cardiovascular Rehabilitation and Fitness, Juntendo University Hospital, Tokyo, Japan; 3 Cardiovascular Rehabilitation and Fitness, Juntendo University Hospital, Tokyo, Japan; 4 Department of Cardiovascular Surgery, Juntendo University Graduate School of Medicine, Tokyo, Japan; 4 Department of Cardiovascular Surgery, Juntendo University Graduate School of Medicine, Tokyo, Japan

**Keywords:** leucine, amino acid, nutrition guidance, lean body mass, grip strength

## Abstract

**Objective:**

Due to the lack of information on the effects of nutritional guidance focused on leucine intake in patients undergoing maintenance cardiac rehabilitation, this study investigated on plasma leucine concentrations, lean body mass, and muscle strength.

**Methods:**

Nutritional guidance, focused on leucine (intervention group) or general nutritional guidance (control group), was provided for six months to patients participating in cardiac rehabilitation. Body composition, grip strength, hematological test results, and diet of both groups were compared before and after the intervention.

**Results:**

Seven patients in the intervention group (53.2 ± 18.2 years) and 7 patients in the control group (58.6 ± 15.3 years) were included. Dietary survey results showed that the six-month intervention significantly (p < 0.05) increased protein intake and estimated leucine intake only in the intervention group. There was no significant difference in the rate of change in plasma leucine concentration between the two groups. The rate of change in lean body mass was significantly higher in the intervention group compared to the control group (p = 0.035). The rate of change in plasma leucine concentration and that in lean body mass was positively correlated only in the intervention group (r = 0.777, p = 0.040), and the rate of change in plasma leucine concentration was also positively correlated with the rate of change in grip strength (ρ = 0.857, p = 0.014).

**Conclusions:**

In the patients undergoing maintenance cardiac rehabilitation, increased plasma leucine concentration by nutritional guidance focused on leucine increased lean body mass without any increasing the training load.

## Introduction

Patients with cardiac disease tend to have a low lean body mass and reduced muscle strength^1)^. Loss of lean body mass and muscle strength can reduce the quality of life of patients with heart disease^2)^, and can worsen heart failure^3)^ and cardiac cachexia^4)^ particularly in older patients. For this reason, it has recently been recommended that exercise therapy in cardiac rehabilitation includes not only aerobic exercise to increase exercise tolerance, but also strength training to maintain and increase lean body mass^2)^. Studies have reported that adding strength training to aerobic exercise in patients with cardiac disease improves muscle strength and cardiorespiratory fitness more than aerobic exercise alone^5, 6)^.

However, for patients with heart disease, it's difficult to set an exercise load produced sufficient exercise effects. Therefore, blood pressure rises and falls at excessively increased the exercise load, acute coronary syndromes and fatal arrhythmias might be develop during exercise, even in patients with preserved left ventricular ejection fraction^7)^.

On the other hands, it has been reported that nutritional intervention in addition to exercise increased lean body mass more than exercise alone^8)^. Therefore, it is considered that nutritional intervention combined with exercise is effective in increasing lean body mass.

Nutritional guidance is the conventional concept of limiting intake such as salt reduction and fat restriction has recently been replaced by the recommendation of nutritional guidance that adjusts the overall diet by adding appropriate nutrients^9, 10)^. For example, nutritional guidance that promotes protein intake may be effective in preventing the loss of lean body mass^11)^. In particular, leucine, an amino acid found in proteins, is the only amino acids that stimulates muscle synthetic pathways^12, 13)^. Therefore, leucine intake is expected to enhance the effects of exercise therapy, especially in patients at risk for sarcopenia. In fact, a study of leucine supplements reported an increase in lean body mass in participants over 50 years of age when they were given leucine supplements in addition to resistance training^14)^. Moreover, older patients undergoing cardiac rehabilitation who consumed a leucine-containing beverage were founded to have increased grip strength, a measure of muscle strength^15)^.

However, no studies have been reported to date examining the effects of nutritional guidance aimed at increasing leucine intake in the daily diet in cardiac rehabilitation patients without relying on dietary supplements. In this study, patients with cardiac disease who participated in maintenance cardiac rehabilitation were provided nutritional guidance focusing on leucine intake over a six- month period, and the effects of this guidance on intake, plasma leucine concentration, lean body mass, and muscle strength.

## Materials and Methods

### Study design

This study was a parallel group comparison trial. Patients were stratified by sex, age, and ability to take pictures and operate machines for a long time and assigned to two groups: one receiving leucine- focused nutritional guidance (intervention group), and the other receiving general nutritional guidance that did not focus on leucine (control group). We also assessed the body composition, grip strength, blood test, and diet of all participants before and six months after the intervention.

### Subjects

Nineteen patients who participated in maintenance cardiac rehabilitation at Juntendo University Hospital between February 2021 and January 2022 were included in the study. The selection criteria were patients (1) at least 20 years old, (2) whose medical condition was stable, and (3) who could obtain permission from their primary physician. We excluded patients with (1) hemodialysis or peritoneal dialysis, (2) decompensated liver dysfunction such as the liver cirrhosis, (3) dysphagia, (4) those taking amino acid supplements, and (5) those unable to regularly attend outpatient cardiac rehabilitation. This study was conducted in compliance with the ethical principles of the Declaration of Helsinki and with the approval of the Ethics Committee of the Juntendo University Hospital (20-078). Written informed consent was obtained from each participant before the study began. This study has been registered in the Japanese Clinical Trials Registry (UMIN000044069).

### Body composition

Height (cm), weight (kg), and lean body mass (kg) were measured using a height meter and bioelectrical impedance method body composition analyzer (780A, TANITA, Tokyo, Japan).

### Grip strength

Long-term decline in grip strength is associated with worse survival and QOL in patients with heart failure^16)^, and grip strength has also been reported to stratify cardiovascular mortality and cardiovascular disease risk in patients with heart disease^17)^. Furthermore, grip strength is said to reflect the muscle strength of the whole body^7)^, and is also used to diagnose frailty^18)^. Therefore, grip strength was measured using a grip dynamometer (T.K.K. 5001: Takei Scientific Instruments Co., Ltd., Kamo, Japan). Measurements were performed alternately twice on the left and right sides, and the maximum value of each side was evaluated using an average of these values.

### Dietary survey

Due to the proven validity of the three-day dietary survey^19, 20)^, nutrient intakes were assessed using pictures and food records over a three-day period, including holidays. In addition, based on previous study^21)^, participants took pictures of their meals together with a predetermined index (9.0 cm x 5.5 cm paper in this study), and noted the amount and type of drinks consumed excluding water. Moreover, participants were instructed by us not to change their diet from their routine diet for three days and the researchers also confirmed this by interviewing the subjects about their diet. Nutritional value calculation was performed by a registered dietitian other than the researchers using a nutritional value calculation software (Excel Add- in Eiyo-plus, KENPAKUSHA Co., Ltd., Tokyo, Japan). A nutritional value calculation software is in line with the Standard Tables of Food Composition in Japan 2020 (Eighth Revised Edition)^22)^ and Standard Tables of Food Composition in Japan 2020 (Eighth Revised Edition)-Amino Acids^23)^.

### Hematological tests

Hematological tests were performed by fasting blood collection. Blood counts and general biochemical tests (hemoglobin (Hb), hemoglobin A1c (HbA1c), uric acid (UA), gamma glutamyl transpeptidase (γ-GTP), total cholesterol (TC), triglycerides (TG), HDL cholesterol (HDL-C), LDL cholesterol (LDL-C), total protein (TP), albumin (Alb), serum iron, ferritin, C-reactive protein (CRP), cystatin C, estimated glomerular filtration rate (e-GFRcys), N-terminal fragment of human brain natriuretic peptide precursor (NT-pro BNP), and amino acid levels were evaluated. Thirty-nine amino acid results were obtained. Nine essential amino acids (leucine, isoleucine, valine, histidine, lysine, methionine, phenylalanine, threonine, and tryptophan) and eleven non-essential amino acids (serine, asparagine, aspartic acid, glutamine, glutamic acid, glycine, alanine, tyrosine, arginine, cysteine, and proline) were evaluated in the results. The values for branched-chain amino acids (BCAA: leucine, isoleucine, and valine) and essential amino acids (EAA) were also calculated.

Blood counts and general biochemical tests other than amino acid analysis were performed in an in-house clinical laboratory, and amino acid analysis was performed in a clinical laboratory (SRL, Inc., Tokyo, Japan) using liquid chromatography- mass spectrometry.

### Nutritional guidance

The intervention group received nutritional guidance at least twice a month, using materials prepared by the researcher, a registered dietitian, with reference to the Standard Tables of Food Composition in Japan 2020 (Eighth Revised Edition) - Amino Acids^23)^. In addition, the frequency of nutritional guidance was set in consideration previous study^24)^ and insurance coverage of nutritional guidance in Japan. In the first nutritional guidance session, we explained the proper intake of EAA (especially leucine) and presented the recommended foods (meat, fish, eggs, soy, and dairy products)^25)^ for the intake of EAA using materials, while instructing the participants to actively consume them. Specifically, we encouraged the consumption of meat with low fat content and fish with high leucine content per gram of protein, including salmon, mackerel, and tuna. In addition, soy and dairy product consumption was encouraged according to dietary balance and renal function. Before the intervention and six months after the intervention, a questionnaire was used to evaluate the frequency and quantity of meat, fish, eggs, soy, and dairy products consumed and to enable participants to reflect on their diet. During the intervention period, the participants were instructed to take pictures of their meals daily during the first three months and at least three days per week after three months, and to report their diet to the registered dietitian.

The control group received standard nutritional guidance based on the Guidelines for Rehabilitation in Cardiovascular Disease^2, 26)^.

### Cardiac rehabilitation

During the intervention period, both groups participated in weekly cardiac rehabilitation (an exercise program) at a specialized cardiac rehabilitation facility. Prior to implementing the exercise program, vital signs were checked, and the program was implemented only if the patient was deemed able to exercise. The exercise program consisted of a warm-up, including stretching, followed by 15 to 20 minutes of aerobic exercise (including various combinations of walking, bicycling, and jogging) and approximately 10 to 15 minutes of light isotonic exercise (including arm curls, shoulder presses and push-ups) and moderate rest, followed by stretching and exercises as a cool-down, for a total of 60 minutes. Aerobic exercise was performed at an intensity equivalent to the anaerobic threshold (AT) measured in the cardiopulmonary exercise stress test or subjective exercise intensity.

### Statistical analysis

After testing for normality using the Shapiro- Wilk test, comparisons were made between groups using Fisher's exact test for sex, disease, and medication status before intervention, and using an unpaired t-test or Mann-Whitney U test for age, body composition, blood pressure, and left heart ejection fraction, and the participation rate of cardiac rehabilitation, depending on whether the date were normally distributed. The participation rate of cardiac rehabilitation was calculated as 100 × the number of sessions actually participated / the number of rehabilitation sessions held. Within- group comparisons between pre-intervention and six months post-intervention were calculated using a paired t-test. In addition, comparisons of each group before and six months after the intervention were analyzed using a two-way repeated-measures ANOVA to identify interactions with and without intervention (group) and before and after intervention (period). In addition, we calculated the percentage change from baseline (pre) in order to variation of change between individuals. The difference between the two groups in the percentage change (⊿) was subjected to an unpaired t-test. Furthermore, the association between the rate of change in plasma leucine concentration, lean body mass, and grip strength was confirmed using Pearson's correlation coefficient or Spearman's rank correlation coefficient, depending on whether the data were normally distributed. Descriptive statistics are presented as mean ± standard deviation, and the significance level was set at p < 0.05. Statistical analyses were performed using SPSS version 28 (IBM Corp., Armonk, NY, USA).

## Results

**Figure 1:**
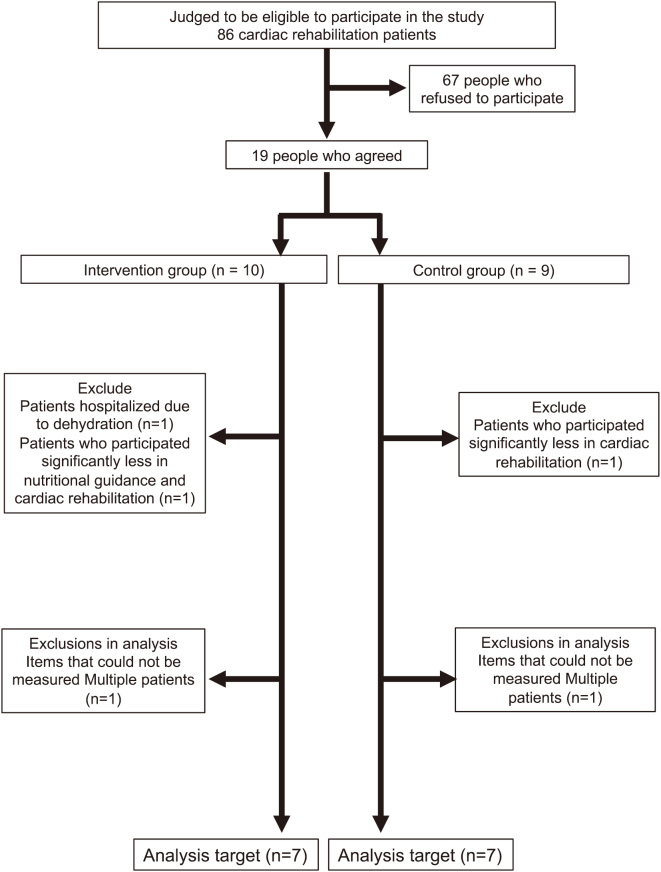
Flowchart of this study

A total of 14 patients in both groups (7 in the intervention group: 53.2 ± 18.2 years, 7 in the control group: 58.6 ± 15.3 years) were included (Figure 1), excluding the patient who was hospitalized due to dehydration without exacerbating heart failure, 2 patients who participated significantly less in nutritional guidance and exercise therapy, and 2 patients who were unable to measure multiple items.

The characteristics of all the analyzed subjects are shown in Table 1. There were no significant differences in the physical characteristics between the two groups. There was also no significant difference in diseases subject to cardiac rehabilitation between the two groups, nor was there a significant difference in left ventricular ejection fraction, a measure of cardiac function. Regarding medication status, two patients in the intervention group were taking diuretics before the intervention, but one of them finished one month after the intervention, and the other reduced the dose immediately after the start of this study. There was no difference between the two groups in β-blocker use or other medications.

**Table 1:** Characteristics of the subject

	Control group (n=7)	Intervention group (n=7)	*P*
Age: Years	58.6 ± 15.3	53.0 ± 18.2	0.559
Height: cm	165.7 ± 7.4	162.3 ± 6.4	0.374
Weight: kg	74.2 ± 14.0	66.9 ± 13.1	0.334
BMI: kg/m^2^	27.0 ± 4.6	25.5 ± 5.2	0.576
Systolic blood pressure: mmHg	122.3 ± 15.0	116.3 ± 19.6	0.532
Diastolic pressure: mmHg	78.7 ± 20.3	76.6 ± 10.3	0.565
left ventricular ejection fraction: %	64.6 ± 6.1	64.4 ± 11.3	0.388
Disease			
Angina pectoris	2	2	1.000
Myocardial infarction	1	2	1.000
Chronic heart failure	1	1	1.000
Auricular fibrillation	1	0	1.000
Postoperative valvular heart disease	2	1	1.000
Postoperative ascending aorta replacement	0	1	1.000
Postoperative congenital heart disease	0	1	1.000
Medication			
Antihypertensive drugs	7	6	1.000
Diuretics	0	2	0.462
Antithrombotic drugs	7	4	0.192
Cardiotonic drugs	1	0	1.000
Drugs for dyslipidemia	6	5	1.000
Diabetes medications	2	0	0.462
Hyperuricemia drugs	1	1	1.000
Amino acid concentration in the plasma			
EAA: nmol / mL	1070.3 ± 127.6	1020.6 ± 123.8	0.474
BCAA: nmol / mL	494.4 ± 70.2	462.3 ± 80.7	0.443
Leucine: nmol/mL	148.2 ± 22.3	138.2 ± 26.7	0.464

Mean ± standard deviation or Number of cases (the number of people) The p-value was calculated using Fisher’s exact test for categorical variables for and t-test or Mann-Whitney U test for continuous variables. Diseases were included for all applicable diseases. BMI; Body Mass Index, BCAA; Branched-chain amino acids, EAA; essential amino acids.

### Participation rate of cardiac rehabilitation

The average participation rate for cardiac rehabilitation was 83.9 % in the intervention group and 90.5 % in the control group, with no significant differences between the two groups.

### Nutrient intake

The weight-adjusted nutrient intakes before and six months after the intervention are shown in Table 2. Only in the intervention group, protein intake increased significantly from 1.00 ± 0.16 g/kg to 1.32 ± 0.31 g/kg after six months compared to pre-intervention (p = 0.003), and confirmed the interaction effect (p = 0.002). Estimated leucine intake also increased significantly from 70.58 ± 16.83 mg/kg to 94.85 ± 26.78 mg/kg after six months compared to pre-intervention (p = 0.004), indicated an interaction effect (p = 0.005). Regarding lipid content, only the intervention group showed a significant increase after six months compared to pre-intervention (p = 0.045), but there was no interaction between the two groups. In the control group, there were no changes in protein, estimated leucine intake, or lipid intake. Energy and carbohydrate content did not change significantly between the two groups. In terms of food intake, meat intake increased significantly in the intervention group after six months compared to pre-intervention (p = 0.033). The intakes of fish, eggs, soy, and dairy products did not change during the intervention period in either group, and no interaction effects were observed.

**Table 2:** Results of dietary survey

	Control group (n=7)				Intervention group (n=7)			Intervention Duration *p*^#^	Group *p*^#^	Interaction *p*^#^
	Before the intervention	After 6 months	p*		Before the intervention	After 6 months	p*			
Energy/ Weight (kcal/kg)	25.12 ± 4.96	25.17 ± 4.05	0.970		23.72 ± 4.47	26.10 ± 5.39	0.143	0.278	0.919	0.301
Carbohydrate content/ Weight (g/kg)	2.97 ± 0.77	2.98 ± 0.53	0.941		3.14 ± 0.95	3.14 ± 0.81	0.984	0.947	0.648	0.970
Fat content/Weight (g/kg)	0.95 ± 0.29	0.91 ± 0.23	0.667		0.84 ± 0.18	1.03 ± 0.22	0.045	0.229	0.982	0.083
Protein content/ Weight (g/kg)	1.10 ± 0.18	0.94 ± 0.18	0.097		1.00 ± 0.16	1.32 ± 0.31	0.003	0.186	0.169	0.002
Estimated leucine content/ Weight (mg/kg)	78.24 ± 21.89	70.09 ± 14.07	0.251		70.58 ± 16.83	94.85 ± 26.78	0.004	0.117	0.402	0.005
Protein mass by food group										
Seafood intake/ Weight (g/kg)	1.20 ± 0.45	1.08 ± 0.62	0.525		0.80 ± 0.48	1.20 ± 0.10	0.050	0.304	0.531	0.069
Meat intake/ Weight (g/kg)	1.10 ± 0.46	0.95 ± 0.32	0.633		1.24 ± 0.60	1.97 ± 1.22	0.033	0.201	0.102	0.063
Egg intake/Weight (g/kg)	0.64 ± 0.50	0.72 ± 0.30	0.627		0.63 ± 0.32	0.94 ± 0.47	0.071	0.105	0.591	0.316
Soy product intake/ Weight(g/kg)	1.83 ± 1.30	0.57 ± 0.66	0.117		0.60 ± 0.45	1.81 ± 2.30	0.718	0.964	0.994	0.032
Dairy product intake/ Weight(g/kg)	1.70 ± 1.62	1.69 ± 0.73	0.972		1.80 ± 1.05	2.34 ± 1.90	0.321	0.493	0.579	0.464

Mean ± standard deviation *The p-value for within-group comparison was calculated using a paired t-test. #The p-value between groups was calculated using a two-way repeated-measures ANOVA. Interactions were assessed by the duration of intervention × groups.

### Hematological tests

**Figure 2:**
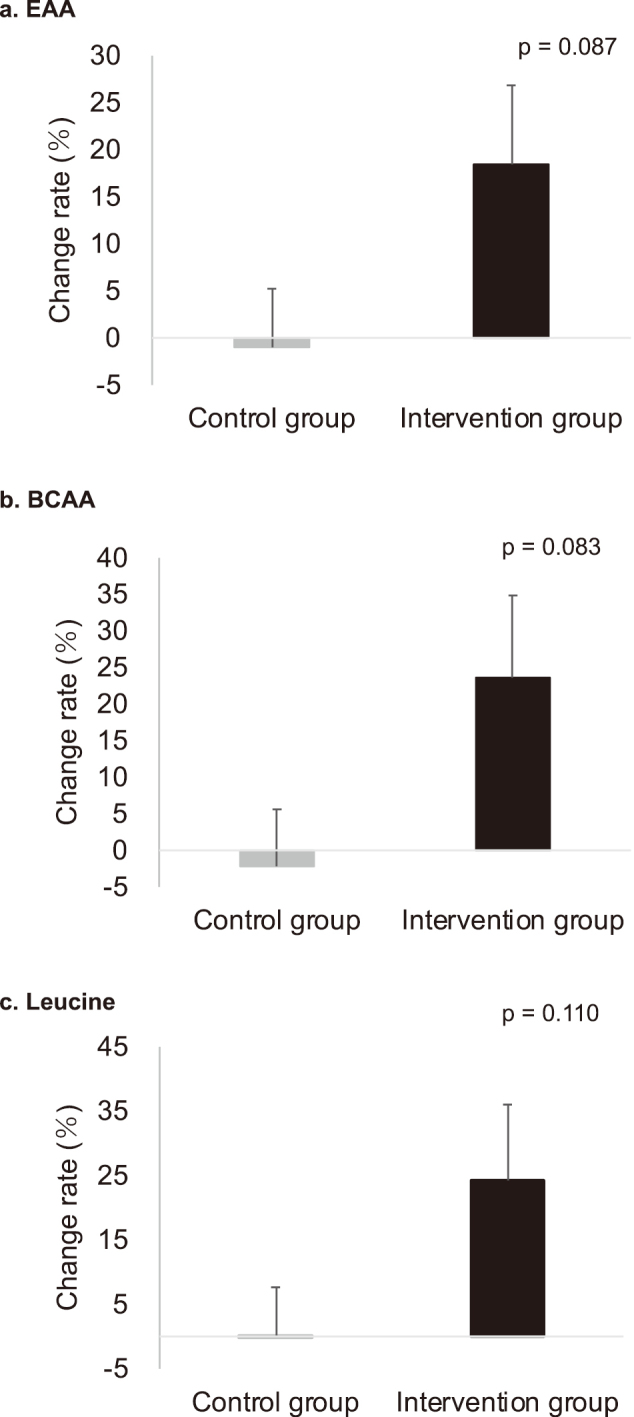
Rate of change in plasma concentrations of EAA, BCAA, leucine The rate of change was calculated as 100 × results after six months/results before intervention-100. BCAA; Branched-chain amino acids, EAA; essential amino acids. The p-value was calculated using an unpaired t-test.

There were no significant differences in the plasma amino acid concentrations between the two groups prior to the intervention. No significant differences were found between the two groups in other blood collection parameters before the intervention. Six months after the intervention, the plasma concentrations of amino acids (EAA, BCAA, and leucine) were not statistically different, and there was no interaction effect, although the values of EAA, BCAA, and leucine were higher in the intervention group (Supplement 1). The rate of change in EAA, BCAA, and leucine levels (100 × results after 6 months/results before intervention- 100) also increased in the intervention group, but no significant difference was observed (Figure 2). On the other hand, histidine showed only significant change among intervention duration (p = 0.002, Supplement 1). However, only in the intervention group, histidine concentration significantly increased from 31.40 ± 39.18 nmol/mL to 79.50 ± 12.66 nmol/mL after 6 months compared to before intervention (p = 0.008). There were no significant differences between the two groups before and after intervention for cystatin C and estimated glomerular filtration rate (e-GFRcys), human brain natriuretic peptide precursor N-terminal fragment (NT-pro BNP), and lifestyle-related parameters (HbA1c, UA, LDL-C, HDL-C, and TG) (Supplement 2).

### Body composition and grip strength

**Figure 3:**
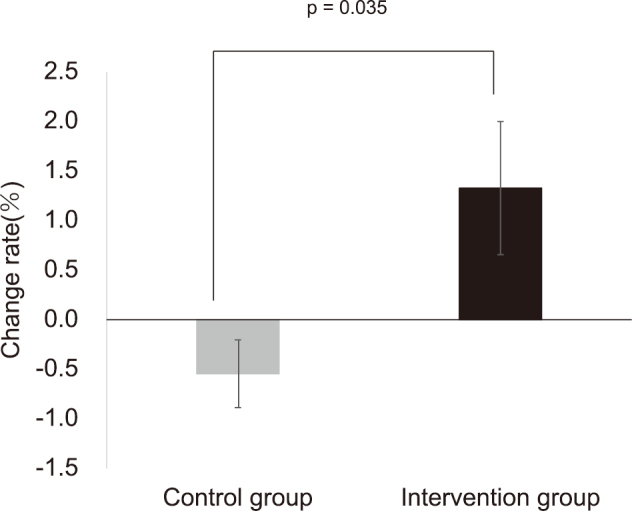
Rate of change in lean body mass The rate of change was calculated as 100 × results after six months/results before intervention-100. The p-value was calculated using an unpaired t-test.

Body weight changed from 73.3 ± 7.3 kg to 72.9 ± 7.0 kg and grip strength changed from 28.7 ± 6.8 kg to 30.5 ± 7.3 kg in the intervention group, but there was no significant change in either group before and after the intervention. However, only in the intervention group, lean body mass increased significantly from 47.3 ± 8.5 kg to 48.0 ± 8.6 kg after six months compared to pre-intervention (p = 0.033) and confirmed an interaction effect (p = 0.033). In the control group, there were no changes in lean body mass (Supplement 3). In addition, the rate of change in lean body mass was significantly higher in the intervention group compared to the control group (p = 0.035, Figure 3).

### Plasma leucine concentration, lean body mass, and rate of change in grip strength

**Figure 4:**
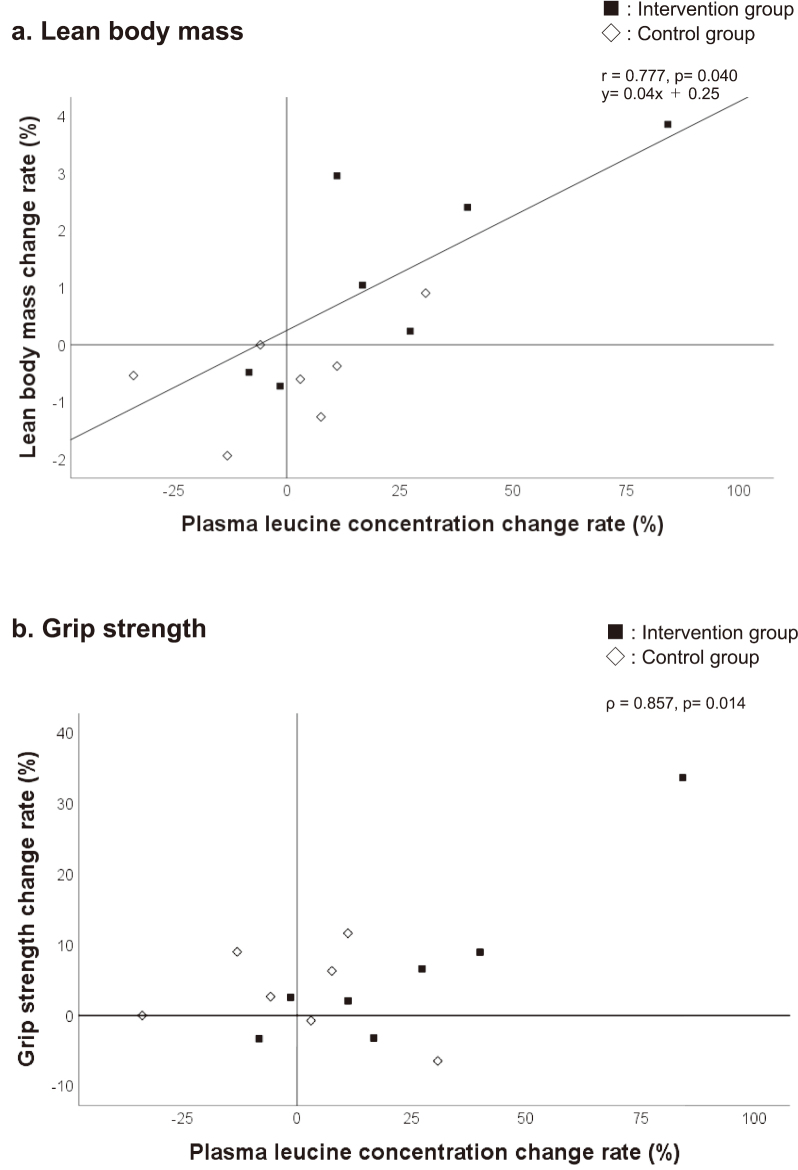
Plasma leucine concentration, lean body mass, and rate of change in grip strength The rate of change was calculated as 100 × results after six months/results before intervention-100. The p-value was calculated using the Pearson product-moment correlation coefficient or Spearman’s rank correlation coefficient. a: Five of 7 participants in the intervention group had elevated plasma leucine concentrations and increased lean body mass after the intervention. Conversely, one out of seven control patients had elevated plasma leucine concentrations and lean body mass after the intervention. In addition, only the intervention group showed a significant positive correlation between plasma leucine concentration and rate of change in lean body mass. b: Four of 7 patients in the intervention group had elevated plasma leucine concentrations and increased grip strength after the intervention. Conversely, in the control group, two out of seven patients had increased plasma leucine levels and lean body after the intervention. In addition, there was a significant positive correlation between the plasma leucine concentration and the rate of change in grip strength only in the intervention group.

The rate of change in plasma leucine concentration and the that in lean body mass were positively correlated only in the intervention group (r = 0.777, p = 0.040, Figure 4). In the intervention group, the rate of change in plasma leucine concentration was also positively correlated with the rate of change in grip strength (ρ = 0.857, p = 0.014, Figure 4).

## Discussion

This is the first study to show that nutritional guidance focused on leucine over a six-month period in patients undergoing maintenance cardiac rehabilitation may increase lean body mass. In addition, these results are also considered important in that they are the first research findings in nutritional guidance.

A previous study reported that skeletal muscle index and grip strength improved after eight weeks of intervention in a group of older post-stroke patients who received a high leucine-containing amino acid supplement in addition to rehabilitation, compared to a group that received only rehabilitation^27)^. Furthermore, it has been reported that in older patients with sarcopenia, 13 weeks of supplementation with leucine, vitamin D, and other nutrients resulted in a significant increase in lean body mass compared with placebo intake^28)^. While previous studies have suggested the potential for increased lean body mass and improved muscle strength with leucine supplementation, this study revealed that similar effects may occur with nutritional guidance focused on leucine when it comes to increasing lean body mass.

On the other hands, no significant changes in grip strength observed as a result of the intervention. However, grip strength increased by approximately 6.3% in the intervention group and 2.6% in the control group after the intervention compared to before the intervention, with the difference being greater in the intervention group. This study had a small number of subjects. Therefore, it is likely that grip strength in the intervention group increased numerically after the intervention compared to the control group, but no difference in the overall average value. Furthermore, it is possible that there were individual differences in results, as some subjects in the intervention group showed a significant improvement in grip strength results after the intervention, while others showed a slight decrease. This may also be relevant to the results of this study.

The results of this study showed that nutritional guidance focusing on leucine increased plasma leucine concentrations in 5 of the 7 patients. Among them, those with the largest increases not only increased lean body mass, but also improved grip strength (Figure 4, Supplement 4). Three patients were able to significantly increase plasma leucine concentrations: case A: Before the intervention, a 22-year-old male's diet consisted of bread and yogurt for breakfast, single dishes such as rice balls and sandwiches for lunch, and an assortment of staples, main dishes, and side dishes for dinner, with lunch being particularly simple. After the intervention, he began to consume more meat and fish at lunch and added soy products to dinner in addition to increasing meat and fish intake. The person in the upper right corner of the intervention group in Figures 4a and 4b is case A. Case B: Before the intervention, a 38-year-old man often ate granola with milk for breakfast, products such as commercial curry and vegetable dishes such as salads for lunch, and staple foods, main dishes, and side dishes for dinner. After the intervention, he added chicken tender and yogurt to breakfast, began consuming meat and fish at lunch, and increased meat and fish intake at dinner. Case C: Before the intervention, a 66-year-old woman consumed a well-balanced diet of staple foods, main dishes, and side dishes at home, but the amount was small overall, and she ate whatever she liked when eating out. After the intervention, she was able to regulate the amount of food eaten at home and choose when eating out. Three patients had been particularly concerned about eating meat and fish before this study. It has also been reported that animal protein is an efficient source of leucine^25)^. In fact, two of three patients listed above increased their meat and fish intake after the intervention, while the other one did not increase after the intervention as fish intake was already secure to begin with, but meat intake did increase. Moreover, the increase in intake of protein-sourced foods in the three participants was greater than in the others, and their diets remained stable throughout the study period. Therefore, establishing a diet that increases intake of protein-sourced foods, especially animal protein, may contribute to increased lean body mass and improved grip strength.

However, despite these changes, increasing dietary leucine intake did not significantly increase plasma leucine concentrations in the intervention group. Plasma leucine concentrations have been reported to be maintained longer with higher leucine intake^29)^. On the other hands, relationships with lipids in the diet^30)^, relationships with dietary fiber^31)^, relationships with dietary forms^32)^, and relationships with lean body mass have also been reported^32)^. Therefore, it is expected that an increase in leucine intake would lead to an increase in plasma leucine concentrations, but there may be variability.

Furthermore, another reason why the plasma leucine concentration did not rise much after the intervention may be that the plasma leucine concentration before the intervention was not low in the intervention group. Although few studies have examined blood amino acid concentrations in Japanese patients with heart disease, it has been reported that the mean blood leucine concentration was 119.6 nmol/L in older patients with heart disease (mean age: 73.1 ± 9.4 years, mean BMI: 22.9 ± 3.2 kg/m2)^33)^ and 125.8 ± 22.7 nmol/L in patients with heart failure with frailty (mean age: 68 ± 12 years, mean BMI: 22.4 ± 4.7 kg/m2)^34)^. In patients without cardiac disease, blood leucine levels were reported to be 140.1 nmol/L in patients with hypertension and other diseases (mean age: 69.1 ± 9.4 years, mean BMI: 24.4 ± 3.4 kg/m2)^33)^, and in persons of similar age and body size as in this study (mean age: 57.0 ± 0.6 years, mean BMI: 27.6 kg/m2) reported 135.5 ± 1.2 nmol/L^35)^. Although there are differences in blood leucine concentration to previous studies, the mean pre-intervention blood leucine concentration of the patients in this study (mean age 53.0 ± 18.2 years, mean BMI: 25.5 ± 5.2 kg/m2), was 138.2 ± 26.7 nmol/L in the intervention group, suggesting that their blood leucine concentrations were not low. The participants in this study had a high BMI and a tendency to overeat before this study, which also likely ensured that they had some leucine intake on a daily basis. This may also have been influenced by the fact that the patients received nutritional guidance during their hospitalization (either during acute or convalescent cardiac rehabilitation) and were less likely to have extreme bias in their diets. Therefore, post-intervention increases in plasma leucine concentrations may have been limited. On the other hand, in these patients, plasma leucine concentrations not only did not increase but also did not decrease after the intervention. Nutritional guidance focusing on leucine intake may have improved the efficiency of leucine intake. Therefore, even if there was a tendency to overeat before the intervention, the efficient intake of leucine would not have led to a reduction, if not an increase, in plasma leucine concentrations after the intervention.

In addition, although no change was observed in plasma leucine concentration, plasma histidine concentrations increased over time in the intervention group. Participants in the intervention group were increased their intake of soy products, which are said to contain antioxidants^36)^, after the intervention compared to before the intervention. The previous studies have reported a negative correlation between the amount of oxygen peroxide, a type of oxidative stress, and blood concentrations of histidine, which is also thought to inhibit oxidation^37)^. Therefore, it is possible that the intake increasing soya products affected increasing plasma histidine concentrations.

This study has several limitations. First, owing to the small number of examinees, it is necessary to consider generalization. However, by providing careful nutritional guidance to the intervention group, we were able to reduce the number of dropouts during the six-month intervention trial. Second, the measurement conditions in this study were not unified. In the future, it will be necessary to standardize the amount of physical activity and meal intake time during rehabilitation at hospitals. Third, participants were provided nutritional guidance using pictures of meals three days, not a daily, a week. Moreover, nutritional guidance in this study was provided at least twice a month, not daily. Although these aspects are generally practiced within the Japanese insurance coverage which effectiveness is well known, in clinical setting, it would be usually discussed about whether the patient complies about meals on other days and whether more frequency of nutritional guidance is more effective. On the other hand, the fact that some patients could change their behavior with nutritional guidance only focusing on content of diet despite such restriction of Japanese insurance system is clinically very important in aspect of patient's behavioral change. Therefore, we would like to consider increasing the number of days for dietary surveys and frequency of nutrition guidance in the future. Finally, in this study, all participants were outpatients and some of them had normal fitness and muscle strength. Therefore, it may have been difficult to generate differences in strength result in leucine intake alone in all participants in this study. The effects of leucine supplementation on muscle strength have been reported, but much of the research has focused on people with low muscle strength^27, 38)^. In the future, approaches to leucine intake should be considered for patients with a maintained ejection fraction who have some fitness and muscle strength, while considering rehabilitation menus.

## Conclusions

In the patients undergoing maintenance cardiac rehabilitation, increased plasma leucine concentration by nutritional guidance focused on leucine increased lean body mass without any increasing the training load.

## Funding

This study was supported by the Joint Research Program (grant number 1412425), the Faculty of Health and Sports Science, and the Institute of Health and Sports Science & Medicine, Juntendo University.

## Author contribution

FK provided their knowledge of sports medicine and revising it critically for important intellectual content. YS provided knowledge of statistical analysis. JN, MD and YS provided cardiac rehabilitation and obtained data acquisition. MY, MT and TM provided knowledge of cardiology. HN provided revising it critically for important intellectual content. KS conceived the original research idea and drafted the manuscript, and provided nutrition guidance and data acquisition and statistical analysis. All authors read and approved the final manuscript.

## Conflicts of interest statement

The authors declare that there is no conflict of interest.

**Supplement 1:** Plasma amino acid profile

	Control group (n=7)			Intervention group (n=7)		Intervention Duration P	Group P	Interaction P
	Before the intervention	After 6 months		Before the intervention	After 6 months			
EAA								
Leucine (nmol/mL)	148.19 ± 22.33	146.07 ± 26.88		138.24 ± 26.68	170.04 ± 45.34	0.147	0.623	0.102
Isoleucine (nmol/mL)	82.34 ± 17.36	75.59 ± 16.76		76.04± 17.71	94.17 ± 28.11	0.341	0.524	0.051
Valine (nmol/mL)	263.86 ± 39.60	254.50 ± 47.50		249.03 ± 38.21	303.54 ± 71.00	0.190	0.459	0.074
Histidine (nmol/mL)	46.06 ± 43.11	85.29 ± 10.08		31.40 ± 39.18	79.50 ± 12.66	0.002	0.395	0.701
Lysine (nmol/mL)	194.16 ± 22.43	193.50 ± 39.40		201.63 ± 43.80	229.26 ± 50.50	0.112	0.304	0.097
Methionine (nmol/mL)	29.51 ± 5.56	28.44 ± 9.26		26.27 ± 3.47	35.07 ± 12.45	0.153	0.657	0.075
Phenylalanine(nmol/mL)	72.91 ± 12.93	71.74 ± 15.25		66.91 ± 15.83	80.96 ± 21.42	0.099	0.847	0.056
Threonine (nmol/mL)	137.51± 35.70	134.34 ± 27.96		128.70 ± 18.77	145.43 ± 23.73	0.419	0.927	0.243
Tryptophan (nmol/mL)	60.57 ± 11.10	60.83 ± 13.40		58.89 ± 10.50	69.11 ± 15.25	0.064	0.609	0.076
NEAA								
Serine (nmol/mL)	108.71 ± 19.90	115.66 ± 29.78		112.04 ± 22.48	123.26 ± 30.41	0.075	0.684	0.654
Asparagine (nmol/mL)	50.11 ± 6.35	50.79 ± 10.99		45.66 ± 5.02	59.73 ± 21.55	0.117	0.625	0.154
Aspartic acid (nmol/mL)	4.87 ± 1.15	4.73 ± 0.86		5.06 ± 1.17	5.50 ± 1.94	0.662	0.465	0.399
Glutamine (nmol/mL)	535.14 ± 61.5	549.01 ± 74.00		581.46 ± 68.23	568.69 ± 101.72	0.956	0.431	0.193
Glutamate (nmol/mL)	63.40 ± 12.52	63.07 ± 16.85		64.29 ± 15.37	64.44 ± 17.70	0.973	0.891	0.922
Alanine (nmol/mL)	431.76 ± 93.52	426.67 ± 105.28		423.90 ± 106.67	447.56 ± 88.61	0.636	0.897	0.467
Tyrosine (nmol/mL)	73.59 ± 11.97	73.31 ± 9.08		69.37 ± 11.45	87.01 ± 27.02	0.104	0.596	0.174
Arginine (nmol/mL)	83.49 ± 18.38	75.93 ± 23.89		88.76 ± 14.08	98.96 ± 21.46	0.853	0.101	0.226
Cysteine (nmol/mL)	31.87 ± 4.52	31.36 ± 6.99		24.66 ± 6.54	24.43 ± 9.27	0.936	0.055	0.835
Glycine (nmol/mL)	203.06 ± 42.92	219.11 ± 50.87		206.61 ± 53.39	204.24 ± 57.15	0.35	0.839	0.215
Proline (nmol/mL)	239.74 ± 90.22	235.09 ± 57.52		194.26 ± 35.08	213.50 ± 51.92	0.614	0.285	0.413
Total BCAA (nmol/mL)	494.39 ± 70.21	476.16 ± 83.21		462.31 ± 80.67	567.76 ± 143.72	0.175	0.505	0.063
Total EAA (nmol/mL)	1070.29 ± 127.63	1050.30 ± 152.01		1020.60 ± 123.83	1207.09 ± 246.86	0.134	0.487	0.069

Mean ± standard deviation
The p-value was calculated using a two-way repeated-measures ANOVA.
Interactions were assessed by the duration of intervention × groups.
EAA; essential amino acids, NEAA; non-essential amino acids, BCAA; branched-chain amino acids.

**Supplement 2:** Blood test values other than amino acids

	Control group (n=7)			Intervention group (n=7)		Intervention Duration P	Group P	Interaction P
	Before the intervention	After 6 months		Before the intervention	After 6 months			
Hb (g/dL)	14.56 ± 1.83	14.69 ± 1.28		14.70 ± 1.34	14.29 ± 1.07	0.490	0.862	0.201
Cystatin C (mg/L)	0.96 ± 0.18	0.96 ± 0.14		0.94 ± 0.08	0.90 ± 0.12	0.293	0.550	0.214
e-GFRcys (ml/min)	80.06 ± 20.11	78.09 ± 15.45		79.63 ± 14.50	85.57 ± 20.73	0.131	0.737	0.760
HbA1c (%)	6.01 ± 0.67	6.01 ± 0.53		5.61 ± 0.29	5.69 ± 0.34	0.394	0.180	0.394
UA (mg/dL)	5.54 ± 1.07	5.04 ± 0.60		5.10 ± 1.38	4.84 ± 1.15	0.097	0.563	0.574
γ-GTP (U/L)	35.29 ± 23.31	35.57 ± 18.35		76.29 ± 90.42	67.14 ± 69.71	0.436	0.265	0.408
TC (mg/dL)	176.71 ± 37.36	175.29 ± 41.77		174.57 ± 47.95	163.00 ± 33.81	0.188	0.739	0.297
TG (mg/dL)	132.86 ± 74.93	159.29 ± 74.47		134.29 ± 38.11	141.29 ± 60.75	0.219	0.798	0.465
HDL-C (mg/dL)	58.00 ± 19.06	57.43 ± 16.34		53.86 ± 9.39	52.29 ± 8.12	0.584	0.533	0.797
LDL-C (mg/dL)	89.57 ± 41.47	87.86 ± 38.55		94.43 ± 46.52	84.71 ± 37.83	0.186	0.969	0.345
TP (g/dL)	6.81 ± 0.28	6.93 ± 0.39		7.04 ± 0.42	6.90 ± 0.13	0.878	0.515	0.182
Alb (g/dL)	4.20 ± 0.26	4.26 ± 0.27		4.41 ± 0.30	4.34 ± 0.14	0.885	0.249	0.208
NT-pro BNP (pg/mL)	213.30 ± 275.72	175.21 ± 200.51		69.08 ± 65.05	80.42 ± 66.48	0.463	0.260	0.188
Serum iron (μg/dL)	97.86 ± 43.20	114.43 ± 64.54		82.86 ± 18.09	93.57 ± 30.65	0.051	0.430	0.650
Ferritin (ng/mL)	175.00 ± 141.50	175.14 ± 150.61		220.29 ± 94.27	217.14 ± 87.50	0.900	0.508	0.891
CRP (mg/dL)	0.10 ± 0.09	0.15 ± 0.21		0.40 ± 0.71	0.10 ± 0.13	0.313	0.461	0.190

Mean ± standard deviation
The p-value was calculated using a two-way repeated-measures ANOVA.
Interactions were assessed by the duration of intervention × groups.
Alb; albumin, CRP; C-reactive protein, e-GFRcys; Estimated glomerular filtration rate (cystatin C), Hb; hemoglobin, HbA1c; hemoglobin A1c, HDL-C; HDL cholesterol, LDL-C; LDL cholesterol, NT-pro BNP; Human cerebral natriuretic peptide precursor N-end fragment, TC; Total cholesterol, TG; triglycerides, TP; Total protein, UA; uric acid, γ-GTP; γ glutamyl transpeptidase.

**Supplement 3:** Results of lean body mass and grip strength

	Control group (n=7)				Intervention group (n=7)			Intervention Duration p ^#^	Group p ^#^	Interaction p ^#^
	Before the intervention	After 6 months	p*		Before the intervention	After 6 months	p*			
	Mean ± SD	Mean ± SD			Mean ± SD	Mean ± SD				
Lean body mass (kg)	53.5 ± 6.9	53.3 ± 7.2	0.334		47.3 ± 8.5	48.0 ± 8.6	0.033	0.343	0.195	0.033
Grip strength (kg)	34.4 ± 7.7	35.3 ± 6.4	0.442		28.7 ± 6.8	30.5 ± 7.3	0.111	0.101	0.185	0.526

Mean ± standard deviation
*The p-value for within-group comparison was calculated using a paired t-test.
#The p-value between groups was calculated using a two-way repeated-measures ANOVA.
Interactions were assessed by the duration of intervention × groups.

**Supplement 4:** Results of plasma leucine concentration, lean body mass, and grip strength in the intervention group (Cases A, B, C, subjects with significantly elevated plasma leucine concentration) and other subjects (Cases D, E, F, G)

	Gender	Age	BMI	Rate of change in plasma leucine concentration (%)	Lean body mass (kg)	Grip strength (kg)
Case A	man	22	17.3	84.3	41.5 → 43.3	24.5 → 32.8
Case B	man	38	29.2	40	62.4 → 63.9	36.3 → 39.5
Case C	woman	66	31.2	27.3	42.0 →42.8	22.8 → 24.3
Case D	man	45	22.3	16.8	47.9 → 48.4	31.5 → 30.5
Case E	man	68	24.8	-1.5	55.4 → 55.0	39.0 → 40.0
Case F	woman	66	22.4	11.1	40.6 → 41.8	24.3 → 24.8
Case G	woman	66	31.1	-8.3	41.6 → 41.4	22.8 → 22.0

The rate of change was calculated as 100 × results after six months/results before intervention-100.
BMI; Body Mass Index.
